# Case Report: Early-Onset Guillain–Barre Syndrome Mimicking Stroke

**DOI:** 10.3389/fneur.2021.525699

**Published:** 2021-02-19

**Authors:** Jing Sun, Yu Gao, Lumei Chi, Qingyang Cao, Zhijie Ning, Guangxian Nan

**Affiliations:** Department of Neurology, China–Japan Union Hospital of Jilin University, Changchun, China

**Keywords:** Guillain-Barre syndrome, stroke, MRI, IVIg, anti-GQ1b

## Abstract

**Introduction:** Guillain–Barre syndrome (GBS) is an acute immune-mediated inflammatory demyelinating polyneuropathy characterized by symmetrical limb weakness and areflexia. GBS can have different clinical manifestations; hence, the initial symptoms are also varied. Here, we describe a rare case of GBS presenting as hemiparesis and cranial nerve palsy, which mimic brainstem stroke.

**Case Presentation:** A 53-year-old man was admitted to the hospital with a 3-h history of left-arm weakness, glossolalia, and right eyelid droop. After admission, his condition suddenly worsened, with quadriplegia, bilateral peripheral facial palsy, bilateral ophthalmoplegia, and other neurological symptoms. Based on the findings from a neurological examination, MRI, cerebrospinal fluid analysis, and nerve conduction study, a diagnosis of GBS was made. He received intravenous immunoglobulin (0.4 kg/day) for 5 days. After 20 days of systematic therapy, his dysphagia, dyspnea, facial paralysis, ocular movement disorder, and leg weakness recovered almost completely, but his arms were still moderately impaired, with a power of 4/5. Fortunately, the patient recovered well without any sequelae after 2 years of follow-up.

**Conclusions:** In patients with an atypical presentation, the diagnosis of GBS is often delayed. With this case report, we intend to highlight the fact that some symptoms mimicking stroke may be a feature of GBS at onset; close observation and timely diagnosis are crucial for clinicians. Neuroimaging is a valuable diagnostic tool in differentiating stroke from GBS.

## Introduction

Guillain–Barre syndrome (GBS) is currently considered to be a severe autoimmune disease that mainly affects a majority of the spinal nerve roots and peripheral nerves and often involves the cranial nerves. The prevalence of GBS is estimated to be 1–3 per 100,000 worldwide, and the disease is more common in males than in females. The initial symptoms are a symmetrical weakness of the extremities, which quickly aggravates and spreads from one end (distal or proximal) to the other, and the trunk and cranial nerves can also be involved; cranial nerve damage is common in bilateral nerve paralysis. Although the most common symptom of GBS is symmetrical paralysis of the extremities, many uncommon initial symptoms, such as unilateral ptosis, vision deficits, urinary retention, unilateral peripheral facial and bulbar palsy, and ophthalmoplegia, have been recorded in detail in the medical literature (1–4). Patients presenting with atypical symptoms pose significant diagnostic challenges to physicians (1). We describe a rare case of GBS presenting as hemiparesis and cranial nerve palsy, which mimic brainstem stroke.

## Case Presentation

**Figure 1:**
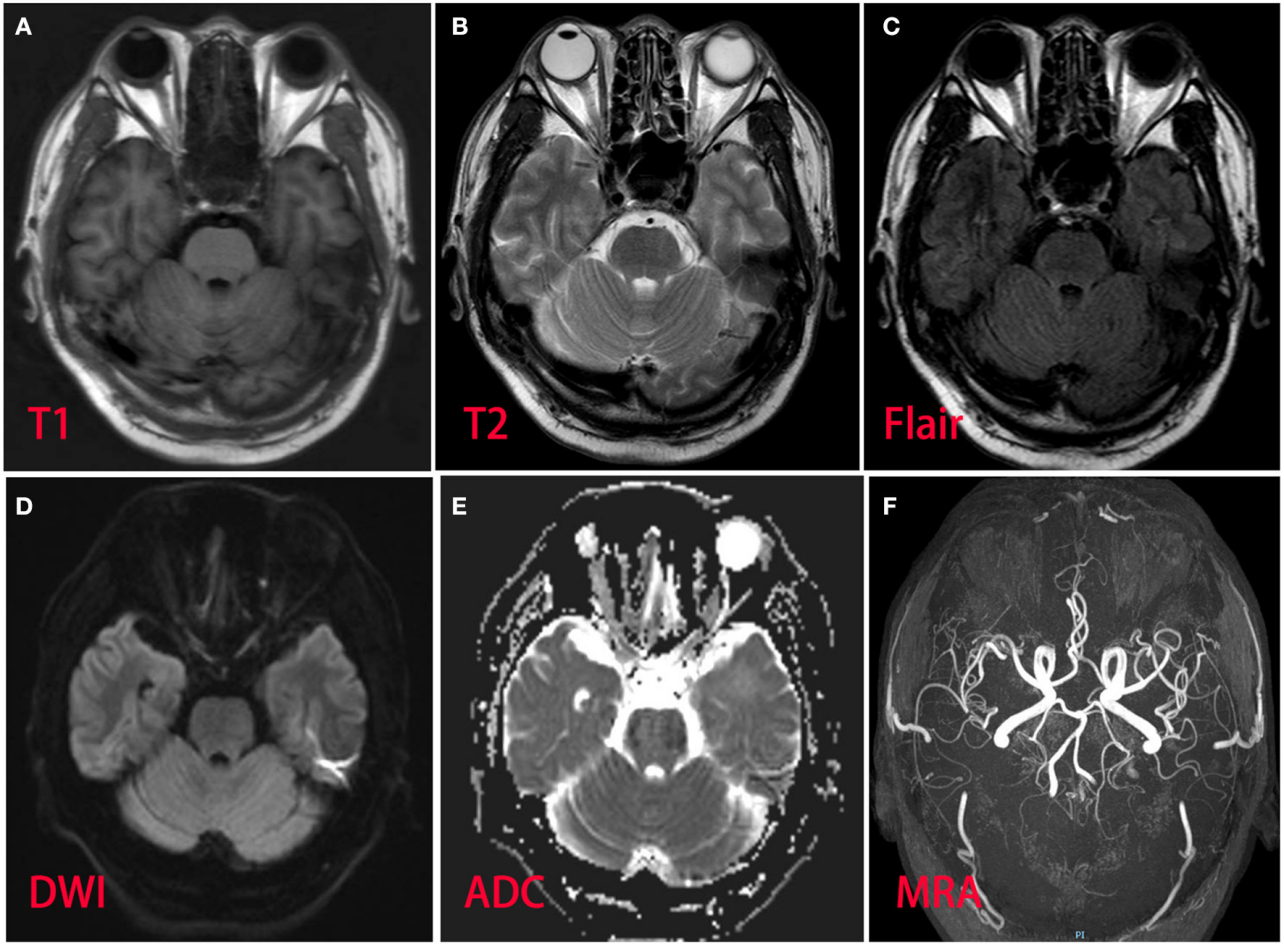
Magnetic resonance imaging and magnetic resonance angiography of the patient brain revealed normal. **(A)** T1 sequence; **(B)** T2 sequence; **(C)** fluid-attenuation inversion recovery (FLAIR) sequence; **(D)** diffusion-weighted (DWI) sequence; **(E)** apparent diffusion coefficient (ADC) sequence; **(F)** magnetic resonance angiography.

**Figure 2:**
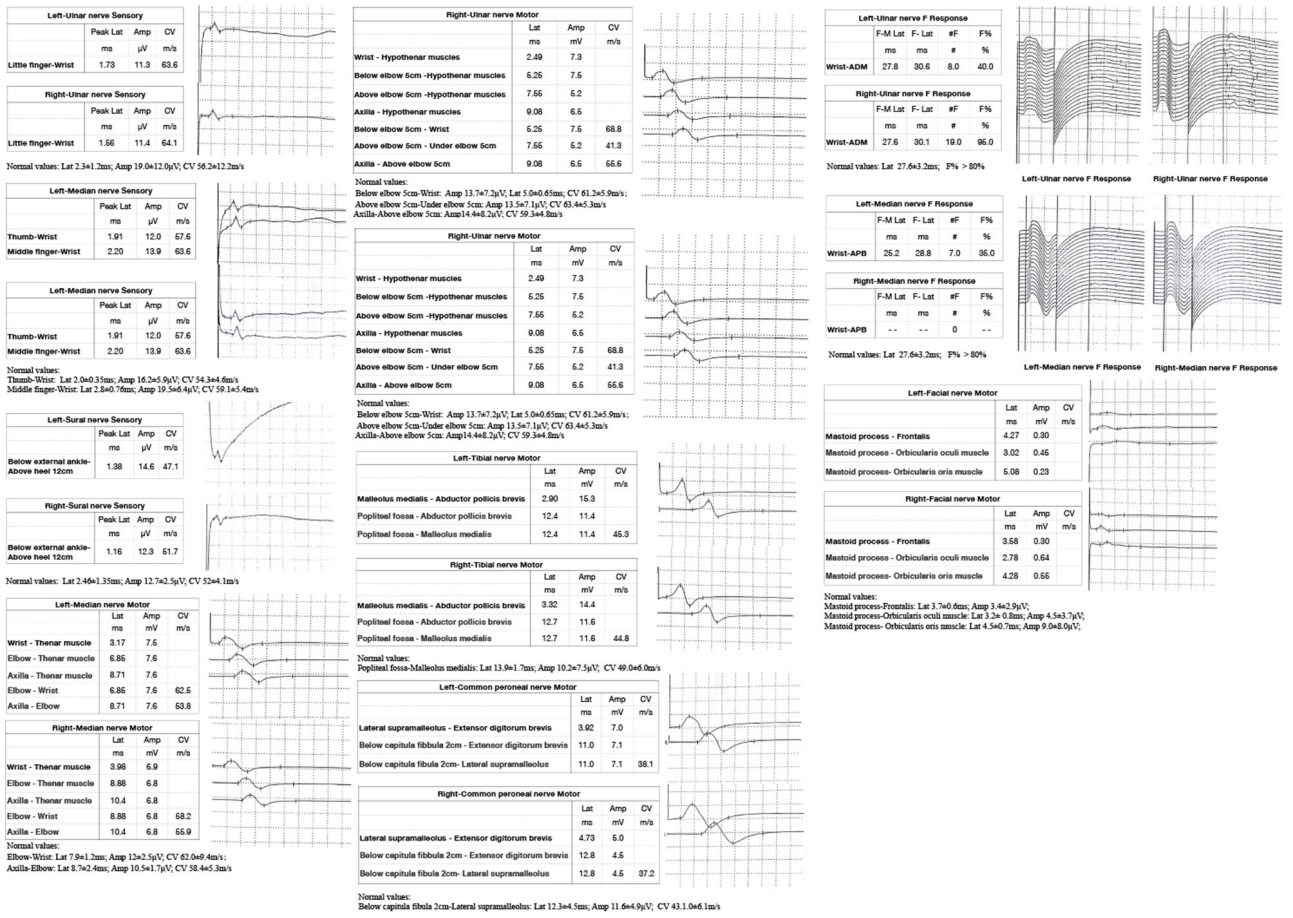
Nerve conduction study (NCS) of the patient showed: the sensory conduction velocity and amplitude of the median nerve, ulnar nerve and sural nerves were normal bilaterally; the motor conduction velocity of bilateral median nerve and right peroneal nerve was normal while the amplitude was reduced; the motor conduction velocity of ulnar nerve was normal bilaterally, while the amplitudes were reduced; the motor conduction velocity and amplitude of the bilateral tibial nerve and left peroneal nerve were normal; the latency and occurrence rate of the F wave in the right ulnar nerve were normal; the latency of the F wave in left median nerve and ulnar nerve was normal, while the occurrence rate was reduced; and the F wave in the right median nerve was absent; the motor conduction velocity of the bilateral facial nerve was normal while the amplitude was reduced.

A 53-year-old man presented with left-arm weakness, glossolalia, and right eyelid droop for a duration of 3 h. He had a sore throat and stuffy nose 10 days earlier. Other than having hypertension and gout, his medical history was unremarkable. None of his family members had experienced similar symptoms. On admission, his vital signs were normal, and his higher mental functions were appropriate for his age. Neurological examination showed dysarthria, right eyelid droop, left facial droop, and a left-held tongue. No nystagmus, ophthalmoplegia, ataxia, or hearing loss was noticed. His muscle strength was 4/5 in the left upper limb (in both the proximal and distal muscles). There was no sensory function deficit. Deep tendon reflexes were present and symmetrical. The results of coordination tests and gait tests were normal, and plantar responses were normal bilaterally. The results of the rest of his physical examination were normal. The results of his brain CT examination were normal. In summary, he was managed as having a posterior circulation infarct. The patient and his family did not agree to intravenous thrombolysis because of the risk of bleeding. Eight hours after he was admitted, his condition deteriorated, with quadriplegia and bilateral peripheral facial palsy. Immediately, cranial MRI with magnetic resonance angiography was performed, but no abnormal manifestations were found (Figure 1). Cervical and thoracic spinal MRI were also performed, and the results were normal. Because of his unremarkable neuroimaging results, GBS became the primary working diagnosis. The following day, he developed bilateral ophthalmoplegia, dysphagia, dyspnea, and numbness in all extremities, and he underwent tracheotomy to prevent a worsening of his acute respiratory failure. Lumbar puncture was performed, and cerebrospinal fluid (CSF) analysis showed that the protein level was 0.87 g/L (normal values: 0.25–0.47 g/L), while the white blood cell count was 5 × 10^6^/L (normal values: 0–8 × 10^6^/L). Anti-ganglioside antibody analysis of the serum and CSF revealed high levels of anti-GQ1b. The blots for other anti-gangliosides (anti-GM1, anti-GM2, anti-GM3, anti-GD1a, anti-GD1b, and anti-GT1b) were negative. Nerve conduction study (NCS) results showed that the amplitudes of the bilateral facial, median, ulnar, and right peroneal (fibular) motor nerves were reduced; the occurrence rates of the F wave in the left median nerve and ulnar nerve were reduced; the F wave was absent in the right median nerve; and the rest of the testing revealed normal results (Figure 2). Unfortunately, the H wave could not be detected in either leg due to the limitations of the patient's posture. According to the NCS results, mild to moderate damage to multiple motor nerves was considered. The patient received intravenous immunoglobulin (0.4 kg/day) for 5 days. Three weeks after admission, at discharge, his dysphagia, dyspnea, facial paralysis, ocular movement disorder, and leg weakness had recovered almost completely, but his arms were still moderately impaired, with a power of 4/5 (in both the proximal and the distal muscles). When the patient was discharged, he no longer needed a ventilator and could breathe normally. The patient had to be flown home, and due to safety concerns, the patient was discharged with a tracheotomy. Fortunately, the patient recovered well without any sequelae after 2 years of follow-up.

The Institutional Review Board of China–Japan Union Hospital of Jilin University approved the study. A signed informed consent was obtained from the patient for publication of this case report and accompanying neuroimages and NCS.

## Discussion and Conclusions

GBS is classically diagnosed by its clinical characteristics, which consist of symmetrical distal limb weakness and/or paresthesia following a mild respiratory or gastrointestinal viral infection. An atypical presentation constitutes a diagnostic challenge for medical specialists given the symptomatic heterogeneity and diverse diagnostic possibilities. There have been very few reports on GBS patients presenting with unilateral limb weakness or facial palsy as onset symptoms (4–11). Among them, the symptoms of some patients were suspected of indicating the onset of stroke (8–11). Brainstem stroke sometimes presents with symptoms similar to GBS. The clinical manifestations of brainstem stroke are varied and depend on the site of occlusion. The appearance of symptoms and signs may be sudden or gradual. The acute sudden onset of limb paralysis and cranial paralysis in this patient led us to first consider the possibility of stroke. However, the patient rapidly progressed to quadriplegia and bilateral nerve palsy, but his neuroimaging findings were normal, which led us to suddenly realize that the patient may have GBS, a disease that can be life-threatening at any time, rather than stroke. Note that this patient's initial presentation was also consistent with the pharyngeal–cervical–brachial (PCB) variant, which is characterized by acute weakness of the oropharyngeal, neck, and shoulder muscles associated with areflexia in the upper limbs (12, 13). Patients with PCB are more likely to have IgG anti-GT1a antibodies, which may cross-react with GQ1b (12). The PCB variant usually does not or only slightly affect the lower extremities, but this patient quickly developed severe paralysis of the lower extremities. Therefore, close observation and timely diagnosis are highly recommended. In this way, the potentially catastrophic consequences of more severe conditions can be avoided. Neuroimaging is a valuable diagnostic tool in differentiating stroke from GBS.

It is known that ophthalmoplegia (usually bilateral) is one part of the triad of Miller Fisher syndrome (14). Interestingly, patients with GBS can occasionally also present with various patterns of ophthalmoplegia in addition to prominent limb motor weakness (15). It has been suggested that ophthalmoplegia in GBS is caused by a similar pathological mechanism as peripheral nerve involvement in the extremities. Kim et al. suggested that ophthalmoplegic GBS occupies a clinical and immunological position between Miller Fisher syndrome and nonophthalmoplegic GBS (16). They found that the most distinct feature in ophthalmoplegic GBS patients was the frequent presence of bulbar palsy and facial diplegia. Most patients with ophthalmoplegic GBS had bilateral ophthalmoplegia with the involvement of the third and sixth cranial nerves during the disease course. It has been reported that anti-GQ1b antibody is closely associated with ophthalmoplegia in GBS (17, 18). GQ1b is a cell-surface component mainly concentrated in the paranodal region of the human oculomotor, trochlear, and abductor nerves (19). Because certain components of pathogens are similar to GQ1b, the body's immune system misidentifies and develops an immune response against GQ1b, resulting in the loss of nerve myelin. Therefore, antibodies to GQ1b are thought to contribute to the pathogenesis of the disease, which was confirmed in this patient. However, bilateral ophthalmoparesis associated with anti-GQ1b antibodies in GBS has rarely been reported. Previous studies have suggested that anti-GT1a antibodies might be associated with bulbar palsy and ophthalmoplegia, but this association was not confirmed in our experience.

Electrodiagnostic studies are not required to diagnose GBS. However, it is recommended that these studies be performed wherever possible, as they are helpful in supporting the diagnosis, especially in patients with atypical manifestations. However, electrophysiological measurements might be normal when performed early in the disease course (within 1 week of symptom onset) or in patients with initially proximal weakness, mild disease, slow progression, or clinical variants (20). In these patients, a repeat electrodiagnostic study 2–3 weeks later can be helpful. Prolonged F latencies have been found most commonly in the posterior tibial nerves (23%) in the lower limbs and in the median and ulnar nerves (18%) in the upper limbs of GBS patients (21), but this patient did not show typical F-wave changes. Note that the reduced amplitudes could be due to axonal loss but also to distal conduction block. This is hard to sort out with only early studies.

GBS is a self-limited disease, but immunomodulatory therapy should be started if the patient is unable to walk independently for 10 m (20). Evidence on treatment efficacy in patients who can still walk independently is limited, but treatment should be considered, especially if these patients display rapidly progressive weakness or other severe symptoms. There are two main immunomodulatory treatment options for GBS patients: intravenous immunoglobulin (IVIg) and plasma exchange (PE). Clinical trials have demonstrated a treatment effect for IVIg when started within 2 weeks of the onset of weakness and for plasma exchange when started within 4 weeks (22, 23). Beyond these time periods, evidence on efficacy is lacking. According to several studies, PE and IVIg were found to be equally effective in the management of GBS (20, 24). However, despite similar efficacy, IVIg is more widely used for GBS due to its higher availability, lack of a need for specialized equipment for administration, and relatively reduced risk for adverse effects. However, the decision to perform PE or IVIg may depend on the patient's clinical circumstance and local factors. Our patient had significant improvement with IVIg treatment. Further comparative studies are needed to evaluate the efficacy of these two treatment options and to determine whether there are any differences in response in each variant of GBS.

There are some limitations of this study, such as a lack of EMG analysis and no second NCS or MRI after tracheotomy. We did not observe abnormal spinal nerve roots or cerebral nerves early on MRI. Because the patient had undergone a tracheotomy, no MRI of the head and spinal cord was reperformed during hospitalization considering the metal's influence on the magnetic field. The family refused a second NCS due to financial reasons. With this case report, we intend to highlight the fact that some symptoms mimicking stroke may be a feature of GBS at onset; close observation and timely diagnosis are crucial for clinicians. These atypical forms of GBS, without classic clinical manifestations, represent a major diagnostic challenge for clinicians. Neuroimaging is a valuable diagnostic tool in differentiating stroke from GBS. When GBS patients display rapidly progressive weakness or other severe symptoms, IVIg or PE should be started immediately to prevent symptom progression.

## Data Availability Statement

The datasets generated for this study are available on request to the corresponding author.

## Ethics Statement

The studies involving human participants were reviewed and approved by Institutional Review Board and Ethics Committee of China-Japan Union Hospital of Jilin University. The patients/participants provided their written informed consent to participate in this study. Written informed consent was obtained from the individual(s) for the publication of any potentially identifiable images or data included in this article.

## Author Contributions

JS wrote the article. YG, LC, QC, and ZN helped draft the article. GN revised the article and supervised this work. All authors have approved the contents of the manuscript.

## Conflict of Interest

The authors declare that the research was conducted in the absence of any commercial or financial relationships that could be construed as a potential conflict of interest.
